# Semen astragali complanati- and rhizoma cibotii-enhanced bone formation in osteoporosis rats

**DOI:** 10.1186/1472-6882-13-141

**Published:** 2013-06-20

**Authors:** Meijie Liu, Gary Guishan Xiao, Peijing Rong, Jiazi Dong, Zhiguo Zhang, Hongyan Zhao, Jingru Teng, Hongxia Zhao, Jinghua Pan, Yan Li, Qinglin Zha, Ying Zhang, Dahong Ju

**Affiliations:** 1 Institute of Basic Theory, China Academy of Chinese Medical Sciences, South Small Street 16, Dongzhimennei, Dongcheng District, Beijing 100700, China; 2 Functional Genomics & Proteomics Laboratory, Osteoporosis Research Center, Creighton University Medical Center, Omaha, NE 68131, USA; 3 Institute of Acupuncture Moxibustion, China Academy of Chinese Medical Sciences, South Small Street 16, Dongzhimennei, Dongcheng District, Beijing 100700, China; 4 Jiangxi College of Traditional Chinese Medicine, Yunwan Road 18, Wanli District, Nanchang City, Jiangxi 330004, China

## Abstract

**Background:**

Growing evidence shows that herb medicines have some anti-osteoporotic effects, the mechanism underlying is unknown. This study aims to investigate the therapeutic effect of Chinese herb supplements on rats that had osteoporosis-like symptom induced by ovariectomy (OVX).

**Methods:**

OVX or sham operations were performed on virgin Wistar rats at three-month old, which were randomly divided into eight groups: sham (sham); OVX control group (OVX); OVX rats with treatments [either diethylstilbestrol (DES) or *Semen Astragali Complanati* decoction (SACD) or *Rhizoma Cibotii* decoction (RCD) or *Herba Cistanches* decoction (HCD) or *Semen Allii Tuberosi* decoction (SATD)]. Non-surgical rats were served as a normal control (NC). The treatments began 4 weeks after surgery, and lasted for 12 weeks. Bone mass and its turnover were analyzed by histomorphometry. Levels of protein and mRNA of osteoprotegerin (OPG) and receptor activator of nuclear factor *κ*B ligand (RANKL) in osteoblasts (OB) and bone marrow stromal cells (bMSC) were evaluated by immunohistochemistry and *in situ* hybridization.

**Results:**

Compared to OVX control, TBV% in both SACD and RCD groups was increased significantly, while TRS%, TFS%, MAR, and mAR were decreased remarkably in the SACD group, only TRS% decreased dramatically in the RCD group. No significant changes in bone formation were observed in either HCD or SATD groups. OPG levels in both protein and mRNA were reduced consistantly in OB and bMSC from OVX control rats, in contrast, RANKL levels in both protein and mRNA were increased significantly. These effects were substantially reversed by treatments with either DES or SACD or RCD. No significant changes in both OPG and RANKL expression were observed in OB and bMSC from OVX rats treated with SATD and HCD.

**Conclusions:**

Our study showed that SACD and RCD increased bone formation by stimulating OPG expression and downregulating RANKL expression in OB and bMSC. This suggests that SACD and RCD may be developed as alternative anti-osteoporotic agents for therapy of postmenopausal osteoporosis.

## Background

Osteoporosis often occurs in postmenopausal women, and older men. Osteoporosis is a systemic skeletal disease characterized by low bone mass and microarchitectural deterioration of bone tissue, leading to an increased risk fragility fractures [1]. Bone remodeling is normal physiological processes of bone that is governed by two counter-processes, bone formation and resorption [2-5]. Currently, available therapies developed for osteoporosis are based on a strategy, which compounds either reduce the rate of resorption or promote bone formation, including bisphosphonates, calcitonin, estrogens, and hormone replacement therapy (HRT). Although these medications are still in use clinically for prevention or treatment of osteoporosis, they often have unwanted side effects. Although HRT remains good treatments for preventing osteoporosis still, these drugs can stimulate endometrial hyperplasia and increase the risk of endometrial carcinoma or breast cancer [6,7]. Therefore, it is urgent to develop an alternative strategy for prevention and treatment of osteoporosis.

Herb medicines have been long used for treatment of osteoporosis in China. According to the theory of the traditional Chinese medicine, kidneys are organs that can usually strengthen the foundation of the essence, regulate development of bones, and stimulate growth of bone marrow. Well-functioned kidneys promote growth of bone marrow, resulting in strength of bones. In contrast, weakly-functioned kidneys often cause development of bone poorly, resulting in an osteoporosis-like symptom, such as lumbar and back pain, leading to an increased risk of bone fragility fractures. Therefore, osteoporosis in China has been treated by using herb supplementation, which it is believed to restore kidneys’ function effectively (Chinese reference may not be provided here).

Use of herb medicines for treatment of osteoporosis has been well documented for long history in China. However, the anti-osteoporotic mechanisms are still unknown. Our previous studies suggest that Chinese herbs increased bone formation by stimulating OPG expression and downregulating RANKL in bone marrow cells [8]. The anti-osteoporotic effects are selective for these herbs. For example, among herb medicines studied in the previous study, only *Radix Dipsaci* and *Pyrola Herb* had significant anti-osteoporotic effects [8]. To develop the most effective herb medicine as an alternative treatment for osteoporosis, in this study we selected additional four herbs, *Semen Astragali Complanati*, *Rhizoma Cibotii*, *Herba Cistanches*, and *Semen Allii Tuberosi*, from 24 Chinese herbs available in Chinese clinics. The criteria for selection of these herb medicines are based on: 1) those potentially effective for therapy of osteoporosis by ancient Chinese medical documents (Chinese reference, not listed here); and 2) those shown mostly effective for therapy of osteoporosis in our previous cell culture and *in vivo* studies (Chinese reference, not listed here).

The OPG/RANKL/RANK signaling pathway has been extensively studied as a molecular model for evaluation of drug efficacy and for understanding molecular mechanism of anti-osteoporosis in animals and humans [9-12]. The mechanism underlying the anti-osteoporotic effects of herb medicines is largely unknown. In this study, we investigated how Chinese herb medicines, especially those “kidney-supplements”, such as *Semen Astragali Complanati*, *Rhizoma Cibotii* decoction, *Herba Cistanches*, and *Semen Allii Tuberosi*, affected bone development, which was examined by measuring the activity of OPG/RANKL/RANK signaling in rats that had osteoporosis-like symptom induced by OVX.

## Methods

### Identification and preparation of extracts

*Semen Astragali Complanati* (SAC) was the dried mature seed of *Astragalus complanatus R*.*Br*. produced in China, and collected by Tianjian Medcial Co. Ltd. Wanfang Branch (Shanxi, China) in March 2007, and identified and authenticated by an expert herbalist at the Institute of Chinese Materia Medica, China Academy of Chinese Medical Sciences (CACMS).

*Rhizoma Cibotii* (RC) was the dried root stalk of *Cibotium barometz* (*L*.) *J*. *Sm*. produced in China, and collected by Zhonghong Herbal Drug Co. Ltd. (Sichuan, China) in March 2007, identified and authenticated by an expert herbalist at the Institute of Chinese Materia Medica, CACMS.

*Herba Cistanches* (HC) was the dried fleshy stem of *Cistanche deserticola Y*. *C*. produced in China, and collected by Taizhou Herbal Drug Co. Ltd. (Zhejiang, China) in March 2007, identified and authenticated by an expert herbalist at the Institute of Chinese Materia Medica, CACMS.

*Semen Allii Tuberosi* (SAT)was the dried mature seed of *Allium tuberosum Rottl* produced in China, and collected by Huarui Medcial Co. Ltd. (Hubei, China) in March 2007, identified and authenticated by an expert herbalist at the Institute of Chinese Materia Medica, CACMS.

All the dried herbs after collection were stored in a dry and sealed container at 4°C to prevent herbs from moisture and moth. SACD, RCD, HCD, and SATD were extracted from the herbs by boiling 300 gram of the dried SAC, RC, HC, and SAT in a 6-litre water at 100°C for 2 h. Each decoction was then concentrated to a final concentration of 1 crude drug gram per milliliter.

The voucher specimens of the plant materials used in this study were deposited in the herbarium of Institute of Chinese Materia Madica China Academy of Chinese Medical Sciences. The deposition number is 37B for *Semen Astragali Complanati*, 13A for *Rhizoma Cibotii*, 10A for *Herba Cistanches*, and 40A for *Semen Allii Tuberosi*.

### Animals and experimental procedures

Ninety-four virgin Wistar female rats (weight 250 ± 20.0 g) were obtained from the Experimental Animal Center of the Academy of Military Medical Sciences (Beijing, China), and were housed in cages in the experimental animal facility at the Institute of Basic Theory of TCM, CACMS. The rats were maintained at 22°C with a 12 h light/dark cycle. During the experiment, the rats were maintained on standard rodent chow (Animal Center of the Fourth Military Medical University, Xi’an, China) that contained 0.9% calcium and 0.7% phosphate; distilled water was available *ad libitum*.

The rats were divided into three groups, including anormal control (NC) group (n = 12), sham control group (n = 12), and a bilaterally ovariectomized (OVX, n = 70) group using the dorsal approach^7^. OVX rats were randomly divided into six groups, including OVX group (n = 12), DES group (n = 12), SACD group (n = 12), RCD group (n = 12), HCD group (n = 11), and SATD group (n = 11). Diethylstilbestrol 0.008 mg/ml dissolved in distilled water was administered intragastrically to the rats in the DES group. The rats of the SACD, RCD, HCD, and SATD groups were treated with SACD 5.6 ml/ (kg body weight day), RCD 5.6 ml/ (kg body weight day), HCD 5.6 ml/ (kg body weight day), and SATD 5.6 ml/ (kg body weight day), respectively. At the same time, the rats of the NC, sham control, and OVX groups were administered with the same volume of distilled water. The treatment started four weeks after the surgery and continued for 12 weeks.

On the fifteenth and the third days before sacrifice, all the rats received tetracyclin 30 mg/kg by intraperitoneal injection. After sacrifice, proximal right tibiae were fixed in 4% paraformaldehyde for 24 h and dehydrated through an ethanol gradient of 80%, 90%, and 100% ethanol, with two days for each step. Dehydrated samples were defatted in xylene for two days before being embedded in a plastic polymer solution I (Methyl Methacrylate Monomer 100 ml + Butyl Methacrylate 35 ml + Methyl Benzoate 5 ml + Polyglycol 1.2 ml), solution II (solution I + Drying Benzoyl Peroxide 0.4 g) and solution III (solution II + Drying Benzoyl Peroxide 0.8 g) with three days for each step. Each undecalcified sample was sliced into two 5 μm longitudinal sections with microtome (Reichert-Jung 2040, Ger). One section was stained with toluidine blue and the other was used for fluorescence morphology observation. Proximal left tibiae were fixed in 4% paraformaldehyde for 24 h and decalcified in a solution of 10% EDTA (PH7.36) at 4°C for three weeks. After that, decalcified samples were dehydrated in 15% sucrose solution for 10 h. Each sample was sliced into 5 μm sections by freezing microtome (OM2563, TBS, CA). Frozen sections were fixed in acetone and were prepared for immunohistochemistry and *in situ* hybridization. All animals were treated according to the *Guide for the Care and Use of Laboratory Animals* and with the approval of the Institutional Ethics Committee of CACMS on animal experiments.

### Immunohistochemical assessment

Frozen sections were mounted on glass slides and used for immunohistochemical analysis. Primary antibodies [anti-rat OPG (1:1000) and anti-rat RANKL (1:1000)] were purchased from Santa Cruz Biotechnology (Santa Cruz, CA, USA). The tissue slides were rinsed in PBS and immersed in 0.3% hydrogen peroxide for 5 min, then, incubated with the primary antibodies for 1h at 37°C, and then rinsed with PBS three times for 3 min. The slides were then incubated with the appropriated unbiotinylated secondary antibody (Zhongshan Goldenbridge Biotechnology Co. Ltd., China) for 30 min at 37°C, followed by incubation with a solution containing DAB (Zhongshan Goldenbridge Biotechnology Co. Ltd., China) for 30 s, then rinsed in running water. After that, the slides were counterstained with Harris hematoxylin and sealed for microscopic analysis. Non-immune goat serum instead of the primary antibody was served as negative control.

All measurements were performed with the QWin image analysis system (Leica Corp., Germany). Five random images within tibiae from two sections were taken, and further analyzed by using zoomed-in field at 400× magnification. We measured the positive stained and total area under each examined field for each section. The positive stain of osteoblasts was calculated by using the percentage of stained osteoblasts over the total trabecula area under each field. The positive stain of bMSC was calculated by using the percentage of stained bMSC over a marrow cavity area excluding trabecula.

### *In situ* hybridization

After warming-up, frozen sections were immersed in a solution of 30% hydrogen dioxide and methanol for 30 min, and incubated in a diluted pepsin solution (3% citric acid) at 37°C for 10 min. After that, the sections were post-fixed in 1% paraformaldehyde for 2 min. Sections were then incubated with the DIG-labeled antisense cRNA probes (see Table 1) at 40°C overnight in a humidified chamber, then followed by performing multiple washes in 4 × SSC at room temperature. Slides were incubated in a blocking reagent for 30 min at 37°C, then incubated with biotinylated anti-digoxin antibody (Sigma, St. Louis, MO, USA) for 60 min, SABC (Wuhan Bpster Biological Technology Ltd, Wuhan, China) for 20 min, and biotinylated peroxydase for 20 min at 37°C, in order, and followed by staining using DAB (Zhongshan Goldenbridge Biotechnology Co. Ltd., China). Finally, sections were covered with glycerol-gelatin and coverslips. For every hybridization procedure, DIG-labeled cRNA sense probes were used as control to rule out nonspecific binding.

**Table 1:** The primer sequence used in the experiments (BORSTER, China)

**Target**	**Primer sequence (5**′-**3)**′
OPG	(1)5′ -TGGAC AACCC AGGAA ACCTT TCCTC CAAAA-3′,
	(2)5′ -TTTGC CTGGG ACCAA AGTGA ATGCA GAGAG-3′,
	(3)5′ -AGAAA TGATA GGGAA TCAGG TTCAA TCAGT-3′.
RANKL	(1)5′-GCCAG CCGAG ACTAC GGCAA GTACC TGCGC-3′,
	(2)5′-GGCCA GGTGG TCTGC AGCAT CGCTC TGTTC-3′,
	(3)5′-TTTAT AGAAT CCTGA GACTC CATGA AAACG-3′.

### Statistical analysis

All values were expressed as means ± standard deviations. All analyses were carried out using the SAS 9.1.3 (Cary, NC, USA). The difference between the groups was analyzed using the ANOVA test followed by the Tukey test.

## Results

### Effects of SACD, RCD, HCD, and SATD on tibia

**Figure 1:**
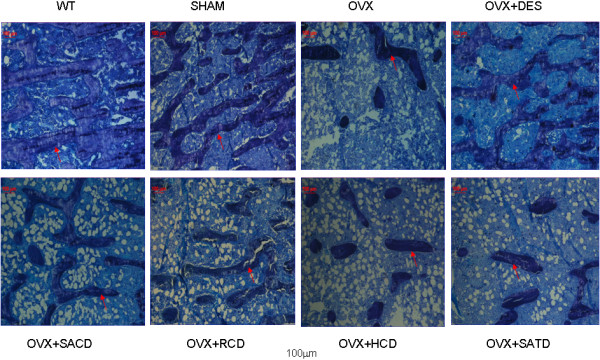
**Effects of herbs on trabecular bone formation.** The undecalcified bone sections were obtained to estimate the effects of Chinese herb suplements on bone. The red arrow indicates trabecular bone. Please see *Methods* section in the text for details.

After three months’ treatment, the right tibia of the OVX rat was harvested, and the undecalcified bone sections were used for bone morphometric analysis (Figure 1). Figure 1 showed that the numbers of the trabecula in tibia from the sham group were similar to those in WT rats, but significantly higher than those in OVX control rats, suggesting that ovariectomy caused loss of trabecular bone. However, OVX-induced effect on bone loss was remarkably reversed in rats treated with DES, SACD, or RCD, but not with HCD or SATD.

Data from analysis of bone histomorphometry are shown in Table 2. Compared to the sham group, the TBV% of the tibia from rats decreased significantly by OVX. However, the OVX effects on TBV% of the tibia were significantly reversed, though not completely reversed, in OVX rats treated with SACD, RCD, or DES, but not with HCD or SATD (Table 2).

**Table 2:** Effects of SACD, RCD, HCD, and SATD on the percentage of trabecular bone volume (TBV%), the percentage of trabecular bone resorption surface (TRS%), the percentage of trabecular bone formation surface (TFS%), 14Hbone mineral apposition rate (MAR), the membrane bone mineral apposition rate (mAR), and the osteoid average width (OSW) of tibia from rats

**Group**	***n***	**TBV%**	**TRS%**	**TFS%**	**MAR(μm**/**d)**	**mAR(μm**/**d)**	**OSW(μm)**
NC	12	28.08 ± 7.26	3.56 ± 1.47	8.23 ± 2.69	1.30 ± 0.18	2.40 ± 0.54	5.65 ± 1.34
Sham	12	27.18 ± 8.78	3.40 ± 1.54	7.40 ± 2.41	1.38 ± 0.16	2.28 ± 0.47	6.20 ± 1.29
OVX	12	8.945 ± 3.04^b^	9.31 ± 2.22^b^	14.54 ± 3.31^b^	1.86 ± 0.23^b^	3.03 ± 0.60^b^	7.77 ± 1.64^a^
DES	12	23.61 ± 4.71^d^	3.28 ± 1.31^d^	7.72 ± 2.66^d^	1.32 ± 0.22^d^	2.15 ± 0.70^d^	6.37 ± 1.42^c^
SACD	12	21.15 ± 4.97^d^	4.71 ± 1.57^d^	9.70 ± 2.29^a, d^	1.55 ± 0.26^d^	2.61 ± 0.31^c^	6.88 ± 1.56
RCD	12	12.10 ± 4.15^b, c^	7.11 ± 1.58^b, c^	14.93 ± 3.50^b^	1.92 ± 0.32^b^	3.09 ± 0.66^b^	7.13 ± 1.82
HCD	11	8.86 ± 2.64^b^	10.31 ± 2.61^b^	12.47 ± 3.92^b^	1.78 ± 0.17^b^	3.05 ± 0.76^b^	7.53 ± 1.86
SATD	11	10.84 ± 3.28^b^	10.06 ± 2.37^b^	12.41 ± 2.91^b^	1.82 ± 0.29^b^	2.96 ± 0.56^b^	6.28 ± 1.82

^a^Compare with sham control group: P < 0.05.

^b^Compare with sham control group: P < 0.01.

^c^Compare with OVX group: P < 0.05.

^d^Compare with OVX group: P < 0.01.

Although TRS%, TFS%, MAR, and mAR of tibia from sham rats were similar to those from normal rats, these indexes were significantly enhanced by OVX compared to sham or normal rats. However, the OVX effects on TRS%, TFS%, MAR, and mAR of the tibia were significantly alleviated, but not abolished, in rats treated with SACD or DES, but not RCD, HCD, or SATD (Table 2). Similarly, OSW of tibia was not significantly different between sham and normal rats, but was sharply increased by OVX. The OVX effect on OSW of the tibia from rats was not changed by treatment with any of herbs (Table 2).

### Effects of SACD, RCD, HCD, and SATD on protein expression of OPG and RANKL in OB and bMSC

**Figure 2:**
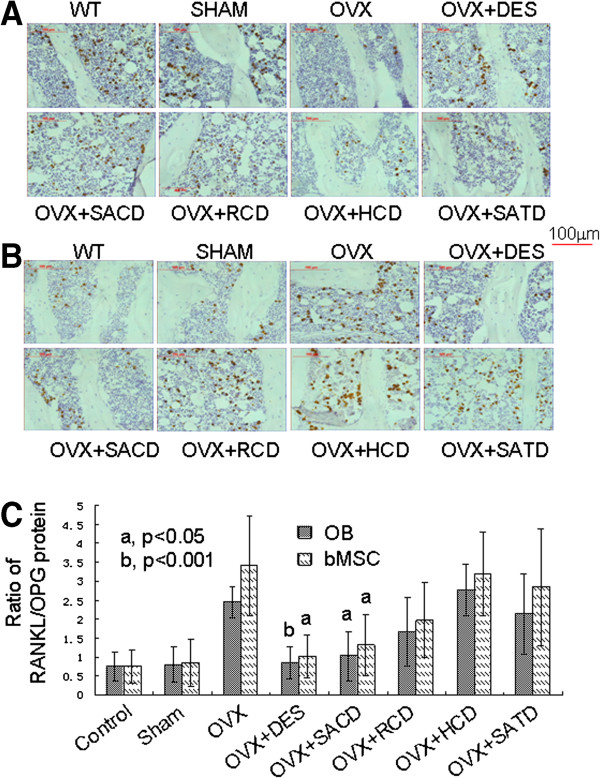
**Effects of herbs on protein expression of OPG and RANKL in osteoblasts and marrow stromal cells from tibia of rats.** Expression level of OPG and RANKL was estimated by immunohistochemical analysis. **A**) OPG expression, **B**) RANKL expression, and **C**) Ratio of RANKL/OPG are shown. Please see *Methods* section in the text for details.

After three-month treatment, expression of bone formation marker OPG in OB and bMSC from rat tibias was examined by immunohistochemistry (Figure 2A). The protein expression of OPG in OB and bMSC from rats in the sham group was similarly highly expressed to that in normal control, but was significantly lower in the OVX rats. However, the OVX effect on expression of OPG in OB and bMSC was alleviated in rats treated with either DES or SACD, but not with HCD, SATD, or RCD (Figure 2A). We calculated the OPG-positive stained area using computer software and showed the results in Table 3. Quantitative measurement of OPG expression (Table 3) confirmed the observation in Figure 2A.

**Table 3:** Effects of SACD, RCD, HCD, and SATD on the protein expression of OPG and RANKL in OB and bMSC from tibia of rats

**Group**	***n***	**The protein expression of OPG in OB**	**The protein expression of OPG in bMSC**	**The protein expression of RANKL in OB**	**The protein expression of RANKL in bMSC**
NC	12	1.66 ± 0.42	7.18 ± 1.72	1.08 ± 0.27	4.77 ± 1.82
Sham	12	1.72 ± 0.52	6.48 ± 1.81	1.17 ± 0.28	4.55 ± 1.75
OVX	12	0.76 ± 0.23^b^	3.06 ± 1.28^b^	1.82 ± 0.41^b^	9.06 ± 2.11^b^
DES	12	1.62 ± 0.38^d, c^	6.24 ± 1.91^d, c^	1.23 ± 0.34^d, c^	5.34 ± 1.54^d, c^
SACD	12	1.51 ± 0.45^d, c^	5.70 ± 1.63^d, c^	1.30 ± 0.30^d, c^	6.40 ± 1.88^a, d, c^
RCD	12	1.25 ± 0.41^a, d^	4.63 ± 1.23^b, d^	1.77 ± 0.36^b^	8.04 ± 2.01^b^
HCD	11	0.72 ± 0.24^b^	3.10 ± 1.05^b^	1.87 ± 0.36^b^	9.14 ± 2.03^b^
SATD	11	0.94 ± 0.30^b^	3.60 ± 1.12^b^	1.75 ± 0.35^b^	8.74 ± 1.84^b^

^a^Compare with sham control group: P < 0.05.

^b^Compare with sham control group: P < 0.01.

^c^Compare with OVX group: P < 0.05.

^d^Compare with OVX group: P < 0.01.

As expected, protein expression of the bone resorption marker RANKL in OB and bMSC from rats in the sham group was similar to that in normal control group. OVX, however, remarkably induced RANKL expression in OB and bMSC from rat tibias (Figure 2B). The OVX effects on RANKL expression in OB and bMSC were significantly reversed in rats treated with either SACD or DES, but not with RCD, HCD, or SATD. The results in Figure 2B were confirmed by quantitative results of RANKL in tibia (Table 3).

The ratio of RANKL/OPG has been used as an index for evaluation of activity of bone remodeling [13]. To help visualize the effects of these Chinese herb supplements on bone remodeling, we re-plotted the data from Figure 2A/B in Figure 2C, of which the expression pattern is similar to that in Figure 2A/B.

### Effects of SACD, RCD, HCD, and SATD on OPG and RANKL mRNA expression in OB and bMSC from tibia of ovariectomized rats

**Figure 3:**
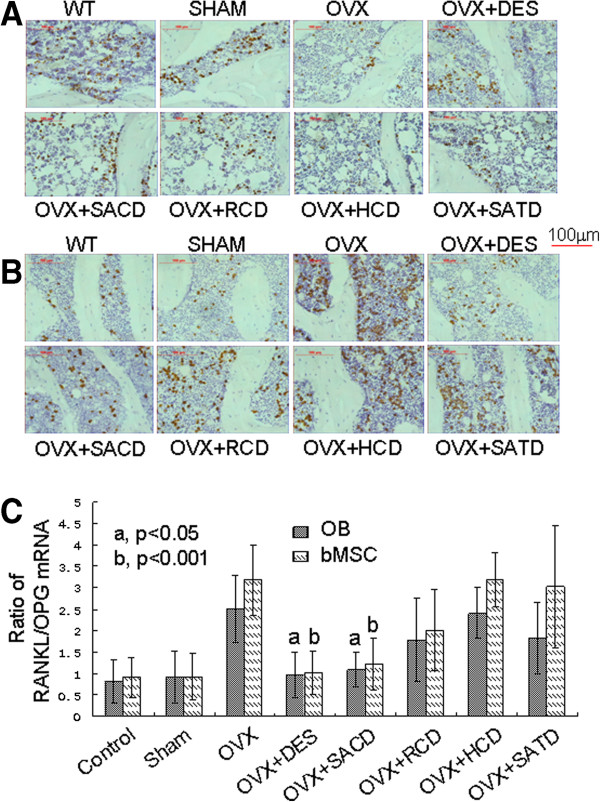
**Effects of Chinese herbs on mRNA levels of OPG and RANKL in osteoblasts and marrow stromal cells from tibia of rats.** Messenger RNA levels of RANKL and OPG in tibia sections were examined by using *in situ* hybridization. **A**) OPG mRNA level, **B**) RANKL mRNA level, and **C**) Ratio of mRNA RANKL/mRNA OPG are shown in this figure. All the experiments were performed three times. Please see the *Methods* section in the text for details.

To further understand whether these medicines affect the mRNA level of OPG and its decoy factor RANKL in OB and bMSC from rats’ tibias, we analyzed mRNA expression levels of OPG and RANKL in tibia sections using *in situ* hybridization (Figure 3 and Table 4). OPG mRNA expression in OB and bMSC from tibia of rats in the sham group was highly expressed, and similar to that in normal control group, but was remarkably downregulated by OVX treatment (Figure 3A). However, OVX effect on OPG mRNA expression was significantly alleviated in rats treated with SACD, RCD, or DES, but not with HCD or SATD. This observation was confirmed by quantitative measurements of OPG mRNA in OB and bMSC from tibia of rats (Table 4).

**Table 4:** Effect of SACD, RCD, HCD, and SATD on OPG and RANKL mRNA expression in * OB and bMSC from rats’ tibia

**Group**	***n***	OPG mRNA expression in OB*	OPG mRNA expression in bMSC*	**RANKL mRNA expression in OB**	**RANKL mRNA expression in bMSC**
NC	12	2.07 ± 0.46	7.33 ± 1.82	1.47 ± 0.52	5.84 ± 1.69
Sham	12	2.15 ± 0.66	7.65 ± 2.19	1.60 ± 0.50	6.07 ± 1.79
OVX	12	1.05 ± 0.40^b^	3.30 ± 1.03^b^	2.41 ± 0.53^b^	9.94 ± 2.16^b^
DES	12	1.98 ± 0.40^d^	6.92 ± 1.65^d^	1.70 ± 0.48^d^	6.26 ± 1.67^d^
SACD	12	1.82 ± 0.36^d^	6.44 ± 1.69^d^	1.83 ± 0.44^d^	6.87 ± 1.71^d^
RCD	12	1.43 ± 0.43^b, c^	5.00 ± 1.46^b, d^	2.18 ± 0.52^a^	8.87 ± 1.91^b^
HCD	11	0.97 ± 0.36^b^	3.28 ± 1.20^b^	2.20 ± 0.60^a^	9.82 ± 1.95^b^
SATD	11	1.28 ± 0.38^b^	3.69 ± 1.47^b^	2.10 ± 0.48^a^	9.51 ± 1.86^b^

^a^Compare with sham control group: P < 0.05.

^b^Compare with sham control group: P < 0.01.

^c^Compare with OVX group: P < 0.05.

^d^Compare with OVX group: P < 0.01.

As expected, RANKL mRNA expression in OB and bMSC from tibia of rats in the sham group remains low and similar to that in the normal control group, but was reduced significantly by OVX treatment (Figure 3B). However, OVX-caused effects on RANKL mRNA expression were remarkably alleviated, although not recovered to the level in normal control, in rats treated with either DES or SACD, but not with RCD, HCD, or SATD (Figure 3B). Results in Figures 3A/B were verified by quantitative measurements of RANKL mRNA in OB and bMSC from tibia of rats (Table 4).

To illustrate the effects of these Chinese herb supplements on bone remodeling clearly, we re-plotted the data from Figure 3A & B (Figure 3C) and found a similar pattern to that in Figure 3A & B.

## Discussion

Although Chinese mherbs have shown an effective therapeutic strategy for Chinese people. the exact mechanism underlying is still unknown. Our previous study and others showed that *Radix Dipsaci* decoction and *Pyrola Herb* decoction enhanced significantly bone formation in OVX rats [8,14]. Our results in this study showed that Chinese herbs SACD and RCD increased bone formation by stimulating OPG expression and downregulating RANKL expression in OB and bMSC.

Resistance to bone fracture depends on its structure and mechanic properties, which are maintained by lifelong bone remodeling [15]. Bone remodeling is controlled dynamically by two-counter balanced processes, bone resorption and bone formation [2,3]. Interruption of the counter-balanced processes leads to an abnormal bone remodeling, resulting further in deterioration of overall skeleton structure. Osteoporosis is highly regulated by many factors including genetic background, age, and sex hormone. The risk of osteoporosis in postmenopausal women is developed incrweasingly due to estrogen deficiency, which causes overexpression of pro-inflammatory cytokines provoking activity of bone resorption [16]. Discovery of OPG, RANKL, and RANK has shed light on understanding of regulation of bone modeling and remodeling [16]. OPG is one of the tumor necrosis factor receptor (TNFR) superfamily members [17], and a soluble glycoprotein secreted by various mesenchymal-derived cells, such as osteoblasts [18] and bone marrow stromal cells [19]. OPG acts as a soluble decoy receptor for RANKL and, thus, decreases bone resorption. RANKL is a TNF-related cytokine expressed by various bone cells, including osteoblasts and their immature precursors [12], activated T lymphocytes and B lymphocytes [20]. RANKL is essential for mediating bone resorption, which stimulates osteoclastogenesis and osteoclast activity by binding to the osteoclast surface receptor [20,21]. RANK is also a member of the TNFR family [22] and is highly expressed by a wide variety of cells, such as osteoclast precursors, mature osteoclasts, B and T lymphocytes, dendritic cells, fibroblasts, and articular chondrocytes, and is considered as a natural receptor of RANKL [21]. The binding of RANKL to RANK leads to the activation of signaling pathways, which regulates function of osteoclast. OPG protects bone from excessive resorption by inhibition of RANKLbinding to RANK [23]. Thus, the OPG/RANKL/RANK signaling pathway is considered as a key cytokine system for controlling bone modeling and remodeling. In this study, we examined the herbal medicines’ effects on bone morphometric indexes and OPG/RANKL expression in OB and bMSC from OVX rat tibia, and found that only SACD, similar to DES treatment, can alleviate the loss of tibia bone mass that was induced by OVX treatment.

Previous studies demonstrated that overexpression of OPG gene in mice resulted in high bone mass and a remarkable reduction in osteoclast number and its activity, while low BMD, increased numbers of osteoclasts and more woven bone were observed in OPG knockout (KO) mice [24]. Injection of recombinant RANKL and OPG to mice caused a rapid and significant increase in bone turnover signals (BTSs) and number of osteoclasts [25]. One OPG injection resulted in a decreased BTS within two hours, and a 50% to 60% reduction in osteoclast numbers within 12 to 24 hours [26]. Our experiments showed that expression of OPG in OB and bMSC from OVX rats treated with either SACD or RCD was significantly higher than that from control, while RANKL expression was much lower than that from control, suggesting that SACD and RCD treatment induced expression of OPG, but decreased RANKL expression, in OB and bMSC.

Intriguingly, we observed that the medicines tested in this study showed similar effects to DES, suggesting that, to some extent, these medicines may contain certain amount of active components, such as phytoestrogen, although the exact active components in these medicines have not been isolated yet. Further functional characterization of these active components from these medicines, especially SACD, may be needed and helpful for understanding the mechanism underlying the medicine-induced effect.

## Conclusions

Data from this study suggest that SACD enhanced bone formation and decreased bone resorption significantly in the treated OVX rats. The mechanism underlying SACD-induced effects in OVX rats may be explained by overexpression of bone formation marker OPG and suppression of bone resorption marker RANKL in OB and bMSC from rats’ tibia.

## Abbreviations

SAC: *Semen Astragali Complanati*; RC: *Rhizoma Cibotii*; HC: *Herba Cistanches*; SAT: *Semen Allii Tuberosi*; SACD: *Semen Astragali Complanati* decoction; RCD: *Rhizoma Cibotii* decoction; HCD: *Herba Cistanches* decoction; SATD: *Semen Allii Tuberosi* decoction; bMSC: Bone marrow stromal cells; MAR: Mineralization rate of trabeculae; mAR: Mineralization rate of bone cortex; OB: Osteoblasts; OPG: Osteoprotegerin; OSW: Osteoid mean width; OVX: Ovariectomy; TBV: Trabecular bone volume; TFS: Trabecular formation surface; TRS: Trabecular resorption surface; DES: diethylstilbestrol

## Competing interests

The authors declare that they have no competing interests.

## Authors’ contributions

ML, carried out rat experiments and drafted the manuscript. GGX and DJ were heavily involved in experimental design, and GGX was also mainly involved in scientific correction of the draft manuscript. PR, ZZ, JD, HZ, JT, HZ, JP, YL, QZ, and YZ were involved in sample collection and measurements of bone morphometry, as well as bone markers. All authors were involved in drafting the manuscript and revising it for critically important content. All authors have read and approved the final manuscript.

## Pre-publication history

The pre-publication history for this paper can be accessed here:

http://www.biomedcentral.com/1472-6882/13/141/prepub
